# CiteSource: An R package for data-driven search strategy development and enhanced evidence synthesis reporting

**DOI:** 10.1017/rsm.2026.10084

**Published:** 2026-04-06

**Authors:** Trevor Riley, Sarah Young, Avery Paxton, Lukas Wallrich, Kaitlyn Hair, Matthew Grainger

**Affiliations:** 1 National Oceanic and Atmospheric Administration Central Library, USA; 2 University Libraries, Carnegie Mellon University, USA; 3 Southeast Fisheries Science Center, National Marine Fisheries Service, National, USA; 4 Birkbeck, University of London, Birkbeck Business School, UK; 5 EPPI Centre, UCL Social Research Institute, University College London, UK; 6 Knowledge Synthesis, Norwegian Institute for Nature Research, Norway

**Keywords:** evidence synthesis, information retrieval, reproducibility, search strategy, systematic searching

## Abstract

Evidence synthesis findings hinge upon well-designed, effective search strategies. When developing these strategies, evidence synthesis teams make multiple decisions (e.g., selecting information sources, developing search string architecture, and picking supplementary search methods) that directly affect the breadth of discovered evidence and thus evidence synthesis outcomes. Despite the number of decisions required when developing search strategies, limited guidance exists to inform these decisions using a data-driven approach. To help address this gap, we developed CiteSource, an R package and accompanying Shiny application, that supports data-driven search strategy development and reporting. CiteSource allows users to assign and retain metadata across three custom fields: *source, label*, and *string* to indicate where the records were found, what method or string was used to find them, and whether they were included after screening. CiteSource allows users to visually map the overlap between sets of records, create data summaries of citation records, and export citation records with the newly assigned metadata. CiteSource’s analysis and visualization outputs can be harnessed for a variety of use cases, such as optimizing literature source selection, honing and understanding the effectiveness of search strings, and evaluating the impacts of literature sources and supplementary search methods. Overall, CiteSource provides a tool for evidence synthesizers to make informed data-driven decisions that boost the efficiency, rigor, and transparency of search strategies and associated reporting.

## Highlights

### What is already known?

- Search strategies in evidence synthesis significantly influence what evidence is considered during screening and what evidence is included in the final synthesis.
- Search strategies largely rely on the researcher’s knowledge/experience and past analysis of information sources and methodologies.
- Optimizing search strings and strategies is time-intensive due to constant changes in the content and functionality of information sources.

### What is new?

- The CiteSource R package and Shiny app assist users in making data-driven decisions to improve search strategies.
- By tracking the provenance of citation data, users can analyze the overlap in citations across information sources and search methods.
- Underlying data can be viewed through various plots and tables, which can speed up the iterative process of validating search strategies.

### Potential impact for Research Synthesis Methods readers

- Researchers can quickly assess the potential impact of information sources and the effectiveness of search strings to optimize strategies based on data.
- Search strategies and string development can be systematically developed and reported to increase the quality and transparency of syntheses.
- Reporting the impact of information sources and search methods can assist in developing further methodological guidance for search strategy development across synthesis methods.

## 1 Introduction

Evidence syntheses, including systematic maps and reviews, require well-designed and effective search strategies to optimize the discovery of relevant evidence. Although a thoughtful and well-designed search strategy is only one of many components of evidence synthesis (e.g., question formulation, literature screening, data extraction, and data synthesis), it can have an outsized impact on evidence synthesis quality.^1^ ^,^ ^2^ This is because search strategies are instrumental in governing the scope and extent of the evidence base that will be discovered.^3^ Optimized search strategies capture relevant articles that match the information needed to address the research questions and aim to balance precision and sensitivity to capture as many potentially relevant articles while maintaining constraints that limit discovery to what is practical and feasible.^4^ ^–^ ^6^ This balance, however, is often elusive, as there is a slippery slope between achieving an optimized search strategy versus one that is more ‘comprehensive’ but may return a high number of irrelevant results that would be impractical and unnecessary for a high-quality synthesis.^7^ ^,^ ^8^ Furthermore, the complexity of searching can lead to errors in accurately applying search strategies.^9^ ^,^ ^10^

When optimizing search strategies, evidence synthesis teams must make multiple decisions that impact the reliability and validity of synthesis findings.^11^ ^–^ ^13^ These include decisions related to selecting information sources (e.g., academic databases, gray literature sources, platform indexes, clinical trial registries, organizational websites, and institutional repositories), determining search string architecture (e.g., selection of terms, use of controlled vocabularies, application of Boolean and proximity operators, truncation, and lemmatization search field selection), and considering which ‘supplementary’ methods to utilize (e.g., citation searching, application of artificial intelligence (AI) platforms, open web searching, contacting stakeholders and subject experts, co-citation searching, and hand searching). A growing body of literature demonstrates that such decisions can impact evidence synthesis findings.^14^ ^–^ ^17^ One study of systematic reviews estimated that 60% of published systematic reviews did not retrieve 95% of relevant articles, largely because the evidence synthesis teams did not search the appropriate combinations of databases, which directly impacts synthesis findings.^18^ Additionally, while supplementary search methods have been shown to yield studies of sufficient quality and relevance not discovered in bibliographic databases,^19^ ^–^ ^21^ the resource-intensive nature of employing various methods can lead to differing opinions as to what methods are justified.^22^

Search strategies are informed primarily by the type of synthesis being conducted, the research question(s) and scope, and the intended use of the synthesis. Strategies are also shaped by external factors, most importantly, time limitations and the availability of resources. Beyond these logistical constraints, the experience, knowledge, and expertise of the research team also play a significant role in shaping search strategies.^23^ While it has been shown that including information specialists in the synthesis process can improve the quality due to their specialized knowledge of information sources and search techniques,^24^ ^–^ ^26^ the information landscape remains challenging. Tacit knowledge can quickly become outdated due to shifts in indexing, industry mergers, and uncertainty around the future funding of government-supported databases and repositories,^27^ ^–^ ^31^ To optimize search strategies and make informed methodological decisions as the landscape changes, information professionals and researchers alike require current and accurate data, specific to their research questions.

Despite the number and impact of decisions required in developing a search strategy, limited guidance exists for how to make these decisions using a data-driven approach. Conducting guidelines that provide detailed information on how to perform a variety of evidence synthesis steps, such as screening and quality assessment, with transparency and minimal bias, typically lack detailed guidance on search strategy development.^5^ ^,^ ^6^ For example, conducting guidelines contain no prescribed way to determine the number of literature sources that should be searched or how to demonstrate that a search strategy has been optimized.^32^ ^,^ ^33^ And, while valuable guidance does exist, including on general strategies,^34^ which bibliographic databases may yield the most relevant articles,^35^ and which citation indices are most appropriate for citation chasing,^36^ such guidance is typically discipline-specific and can quickly become outdated as the information landscape shifts and new technology, resources, tools, and methods are developed.^37^ ^–^ ^40^

To help address the gap in quantitative search strategy guidance, we developed CiteSource, an R package and accompanying Shiny application, that supports data-driven search strategy development and reporting. Our goal was for CiteSource to serve as a tool for ensuring effective search methods, increasing the efficiency of search string development and testing, improving search strategy optimization, boosting search strategy transparency and reproducibility, and conducting analyses to assess outcomes of search strategy decisions. Overall, CiteSource provides real-time analysis functions, allowing evidence synthesizers to make data-informed decisions that boost the efficiency, rigor, and transparency of search strategies and associated reporting, which in turn facilitates comprehensive documentation consistent with established reporting standards (e.g., PRISMA-S and ROSES).

CiteSource adds to the growing suite of R packages designed to support search strategy development for evidence synthesis. For example, litsearchr is an R package and Shiny app that uses keyword co-occurrence networks to identify relevant search terms and phrases from a corpus of initial search results, aiding the term harvesting step of search strategy development.^38^ It also provides an automated approach to the construction of a Boolean search strategy from a list of key terms and a tool to compare a set of search results to a list of known benchmark articles. searchbuildR is another, more recent example of a search strategy construction tool.^39^ searchbuildR uses term frequency analysis to suggest keywords, including a view of their context within the abstracts, and PubMed identifiers to generate lists of medical subject headings (MeSH) terms. Users can construct a random set of records for search strategy development and a set for testing the search strategy, and can also test the consequences of widening or narrowing the distance between keywords when using proximity operators. While such tools support search term harvesting and search strategy construction, CiteSource is the first R package designed to test full search strategies across different sources and methods.

This paper introduces the CiteSource R package and accompanying Shiny application. We begin by outlining CiteSource’s core functionalities and the features designed to empower researchers in their search strategy development. Subsequently, we present practical use cases to demonstrate CiteSource’s utility in optimizing information source selection, refining search strings, and assessing the impact of various search methods. The paper concludes with a discussion of CiteSource’s broader implications for enhancing evidence synthesis methodology, promoting reproducibility, and supporting the training of information specialists and researche rs (Box 1).

Box 1.
Definitions of terms used throughout the paper are provided in Box 1.

**Search strategy:** The overall plan and application of search methods designed to identify and retrieve relevant evidence for an evidence synthesis project. Strategy encompasses the methods and techniques used to identify and retrieve relevant evidence.

**Search method:** An approach used to discover relevant records. This includes, but is not limited to, searching bibliographic databases/platforms, citation searching, use of AI platforms, open web searching, and contacting experts.

**Search technique:** A specific style/process applied within a search method (e.g., Searching title/abstract vs. title/abstract/keywords in bibliographic databases).

**Search string:** The combination of keywords, controlled vocabulary, Boolean operators, proximity operators, truncation/wildcards, and search fields, and any applied filters to query an information source (typically a bibliographic database/platform).

**Syntax:** The set of rules, commands, operators, symbols, and structure used to formulate a query that can be correctly interpreted by a particular search engine, database, or information source.

**Record:** A bibliographic entry, which represents a single citable work (e.g., a journal article, a book, a book chapter, a report, etc.).

**Set of records:** A strictly defined group of records (e.g., results from Web of Science, results from a variation of a string, and records included after screening). In CiteSource, the source, label, and string fields can be used to define sets of records.

**Unique record:** A record that has been found to have no duplication across all records being analyzed.

**Duplicate record:** A record that, when compared to other records, has been found to point to the same citable work.

**Distinct record:** A record that has been identified as unique within a set of records (e.g., when two search strings from one database are combined and deduplicated). Distinct Records may be found to be unique or duplicates when compared to a larger set of records (e.g., when comparing search results from Scopus and Web of Science).

## 2 Functionality

CiteSource is available as a downloadable R package, as well as a Shiny application. Here, we take a stepwise approach to describe the functionality of CiteSource using the Shiny Application. Detailed CiteSource vignettes that walk through use cases are listed under the ‘articles’ tab on the CiteSource website (https://www.eshackathon.org/CiteSource/)

1. **Upload citation files:** Users upload multiple citation files (.ris, .txt, or .bib) containing search results or records of included articles. The package also provides an option to re-upload .ris and .csv data that were previously exported from CiteSource, allowing users to reproduce all plots and tables with a single file.
2. **Assign custom metadata fields:** After uploading citation files, users assign custom metadata across three fields: ‘cite source’, ‘cite label’, and ‘cite string’ referred to in this paper and in the shiny app as *source*, *label*, and *string*.
  - ***Source*:** used to tag citations with metadata related to their source (e.g., Web of Science, and Scopus) or the method used to find them (e.g., citation searching, numbered search string). *Source* is a free-text field.
  - ***Label*:** used to tag citations with information related to their associated screening phase. The label field requires one of three terms: ‘search’, ‘screened’, or ‘final’. All plots/tables require at least one file to be labeled as ‘search’, no other terms in the label field are permitted.
    - **Search**: indicates the citations are associated with initial search results (including benchmark articles).
    - **Screened**: indicates that the citations were included after title/abstract screening.
    - **Final**: indicates that the citations were included after being screened at the full-text level and included in the final synthesis.
  - ***String*:** used to further differentiate sets of records. While the source/label fields alone can handle most use cases, the string field can be used to record other supplementary information a user may want to retain for analysis, or to further differentiate string variations (e.g., String1-narrow, String1-broad, String2-narrow, etc.) *String* is a free-text field.
3. **Deduplicate citations:** After tagging uploaded datasets, users must initialize the automated deduplication process. At the core of its functionality, CiteSource utilizes the Automated Systematic Search Deduplicator (ASySD) package to identify duplicates and automate the deduplication process.^41^ ASySD identifies duplicates by running multiple rounds of comparisons to find potential matches based on shared metadata. Once potential pairs are identified, Jaro–Winkler similarity scores are calculated for each field and used together in over twenty combinations to classify pairs as ‘true duplicates’ when they meet specific thresholds (e.g., pages > 0.8 + volume > 0.8 + abstract > 0.9 + author > 0.5 + Journal > 0.6). Once automated deduplication is complete, users have the option to manually review records that were flagged due to their similarity, but which did not meet the threshold for automatic classification as duplicates.
4. **Analyze data:** Once deduplication is complete, users can generate a number of plots and tables to analyze the data (Tables 1 and 2). For a variety of use cases, examples of these are covered in the section on use cases.
5. **Export data:** Finally, users can export record metadata as .csv, .ris, and .bib files. Custom metadata is retained as part of the exported data. Exported data can later be reimported into CiteSource, these data can also be made available when reporting synthesis findings for added transparency and to provide an opportunity for aggregated analysis when combined with data from other syntheses.

**Table 1:** CiteSource can produce multiple visualizations to help analyze search strategy outcomes Table 1 Long description.

Plots	Function
Heatmaps	Visualize the number and percent of overlap between two sources, methods, or techniques
Upset plot	Visualize the number of unique and overlapping records across multiple sources, methods, or techniques
Bar chart	Visualize the number of unique and overlapping records for each source, method, or technique across screening phases

**Table 2:** CiteSource can produce a number of tables to help analyze and report search strategy outcomes Table 2 Long description.

Tables	Function
Record summary table	Displays the number of unique/overlapping records, percent of unique records contributed, and percent of records unique to each source, method, or technique
Precision sensitivity table	Overview of the number of records that were included across screening phases for each source/method. Includes each source/method precision and sensitivity/recall calculation
Record level table	Interactive table that allows users to search full citations, sort based on source/method association, and link directly to records via DOI or URL

## 3 Use cases

CiteSource has a number of use cases that generally fall under two areas: optimizing search strategies and analyzing impact. Optimizing search strategies can include source selection and string development, as well as some aspects of search method selection, such as whether or not to include certain ‘supplementary’ methods like citation searching. Using the built-in visualizations and tables, typically iterative multi-step processes can be compressed into a single analysis step, saving users time in testing and validating strategies. For example, differences in search results from changes in the structure of Boolean operators, the use of stemming, lemmatization, near-term operators, or the use of different search fields can be compared and contrasted in a single plot. When using CiteSource to optimize a search, the package is applied during protocol development. Conversely, when used to determine the impact of a specific information source or method, data are analyzed only after the final inclusion/exclusion decisions are made, as it is necessary to see the overlap with the included records. By providing researchers with an understanding of the contribution of sources and methods to a given review, this type of analysis and reporting can provide much-needed data for evidence-based methodological approaches in updates and future syntheses on related topics.

### 3.1 Search strategy optimization

Search strategies are informed primarily by the type of synthesis being conducted, the research question(s), and the intended use of the synthesis. Strategies are also shaped by key external factors, such as time constraints, the availability of information resources and screening tools, as well as the experience, knowledge, and expertise of the research team.^23^ ^,^ ^33^ While librarians and information specialists typically provide guidance on source selection and best practices for searching, the information landscape constantly changes with shifts in publishing and indexing practices, merging of information aggregators and publishers, and uncertainty around federally sponsored databases and repositories; all pose significant challenges for search strategy development.^27^ ^–^ ^31^

#### 3.1.1 #1 Information source selection

A primary application of CiteSource involves facilitating data-driven information source selection within the review process. Through the systematic tagging of search results with ‘source’ metadata, CiteSource enables researchers to determine the degree of citation overlap and uniqueness among information sources. This analysis illustrates which sources have the greatest potential for review versus those that may be considered excessive or potentially insignificant due to substantial duplication. We broadly consider ‘Information sources’ to encompass contributions from distinct methodologies, such as citation chasing, in effect, treating a single paper as a source of information. Consequently, by integrating the assessment of both information sources and methodological approaches, researchers can attain a holistic visualization of the collective performance of their comprehensive search strategy.

**Figure 1:**
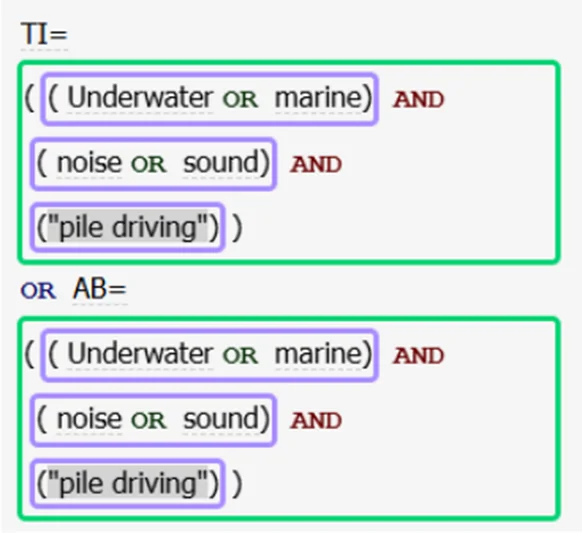
Example Search String is a simpler approach combining the three PEO concepts within each search field. This figure shows strings in Web of Science syntax (visualized in https://www.medsyntax.org).^42^ String syntax was adapted to meet the syntax requirements for each database. Full strings can be found in the supplementary file.

As an example of how CiteSource can be applied to guide the selection of information sources, we can consider the following scenario. A research team is investigating the ecological consequences of acoustical disturbances originating from pile driving activities in marine settings. The selection of appropriate information repositories may require the inclusion of discipline-specific databases, for instance, ProQuest’s Aquatic Sciences and Fisheries Abstracts (ASFA) and Oceanic Abstracts (OA), as well as multidisciplinary platforms, including Clarivate’s Web of Science (specifically, the Science Citation Index Expanded—SCIE), Elsevier’s Scopus, and Digital Science’s Dimensions. Furthermore, the team believes that academic theses and dissertations may be valuable and decides to include specialized databases such as ProQuest’s Open Access Theses and Dissertations (OATD). To initially evaluate the potential coverage and overlap of these sources, a relatively broad ‘naive’ search string is developed to capture a wide array of potentially relevant records. This string (Figure 1; Web of Science syntax) was subsequently adapted and applied across the six information sources. The results were then uploaded to CiteSource for comparative analysis, with each set of records distinguished by their assigned ‘source’ metadata.

**Figure 2:**
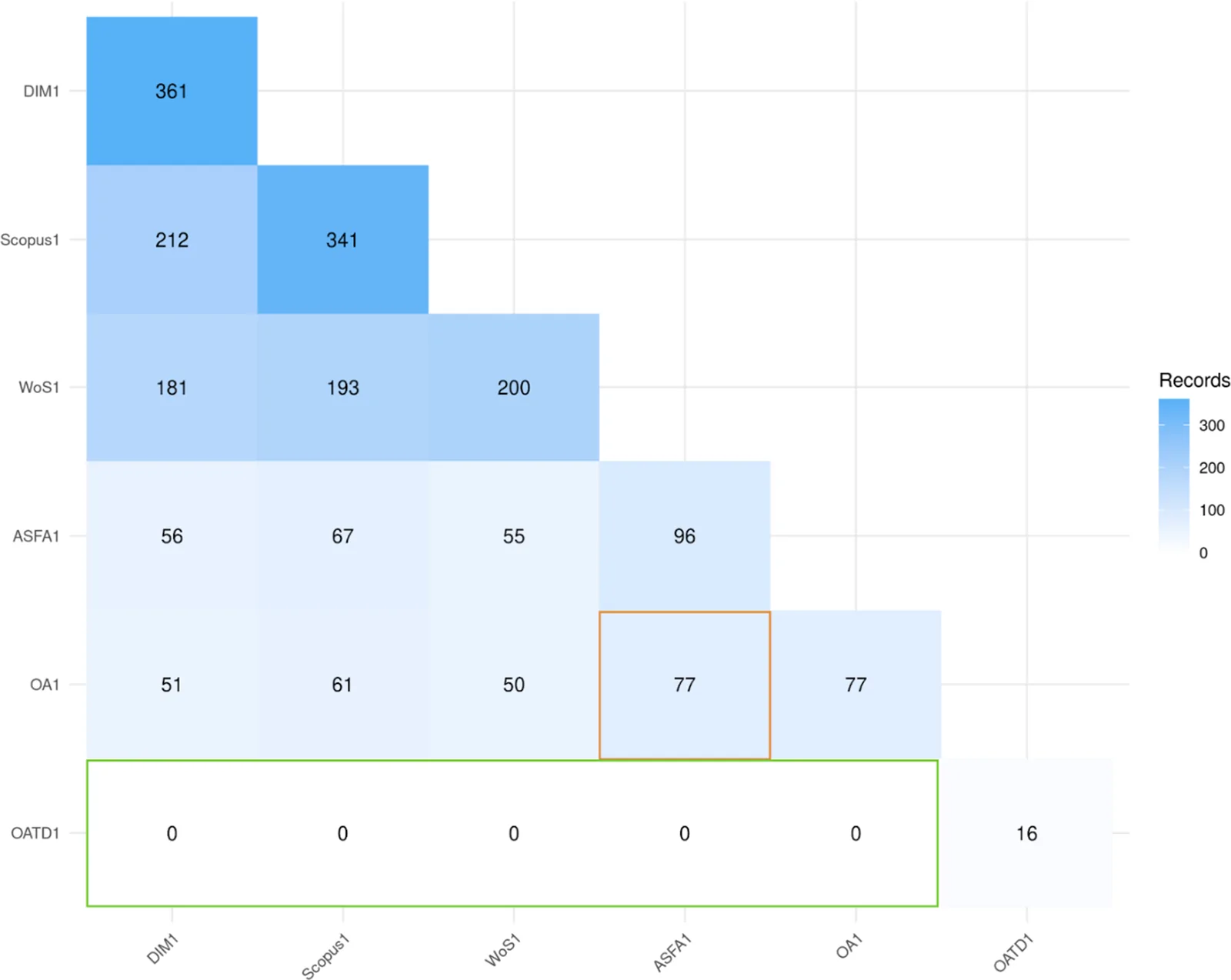
A heatmap showing the six sets of records uploaded and the number of overlapping records between each set. In this example, all 77 records from Ocean Abstracts (OA) are found in Aquatic Sciences and Fisheries Abstracts (ASFA), highlighted here in orange. The plot also shows that none of the 16 records from ProQuest’s Open Access Theses and Dissertations (OATD) were found in any of the other sources, highlighted here in green. Figure 2 Long description.

Once automated deduplication is complete, the team can quickly assess the overlap between each source. In this example, the heatmap (Figure 2) clearly shows us that all 77 records that were found in Oceanic Abstracts (OA) were also found in Aquatic Sciences and Fisheries Abstracts (ASFA). This overlap is explained by the fact that Proquest’s Oceanic Abstracts is included within the ASFA database while maintaining its own scope and the ability to search the database independently. While this overlap was included by design in this example, it provides an example of how CiteSource can be used to identify sources that may not provide unique content. In this case, the team (who may or may not have been knowledgeable about the ASFA/OA relationship) can use these data to make a data-informed decision to drop Oceanic Abstracts as a source. Simultaneously, recognizing a highly unique source like OATD might prompt consideration of exploring additional specialized repositories for theses and dissertations to ensure comprehensive coverage of this document type.

This example highlights CiteSource’s capacity to support data-driven information source selection, as illustrated by the potential findings of redundancy between ASFA and Oceanic Abstracts and the novel records contributed by OATD. CiteSource facilitates the strategic inclusion of sources offering unique value and the exclusion of those providing largely redundant content, thereby optimizing the efficiency and effectiveness of evidence-gathering efforts from the outset.

#### 3.1.2 #2 Search string development

Search strings can range in complexity based on several factors, including the research topic, scope, database/platforms being searched, etc. Testing and iterating search strings can be time-consuming, due to differences in syntax across platforms and the need to compare the results of various techniques. CiteSource assists users in systematically evaluating and refining search strings. This process involves not only optimizing retrieval to enhance sensitivity and precision but also identifying and rectifying potential critical errors in syntax or logic,^9^ all while speeding up the iterative analysis. By enabling the distinct tagging of search result sets (e.g., by string version or database) and the subsequent analysis of overlapping and unique records, users can quickly assess multiple iterations of a search string. Examples of such assessments include evaluating:

- the impact of including or excluding terms
- variations in syntax
- different combinations of strings
- different approaches to Boolean architecture
- the use of different search fields
- the effectiveness of capturing benchmark articles

**Figure 3:**
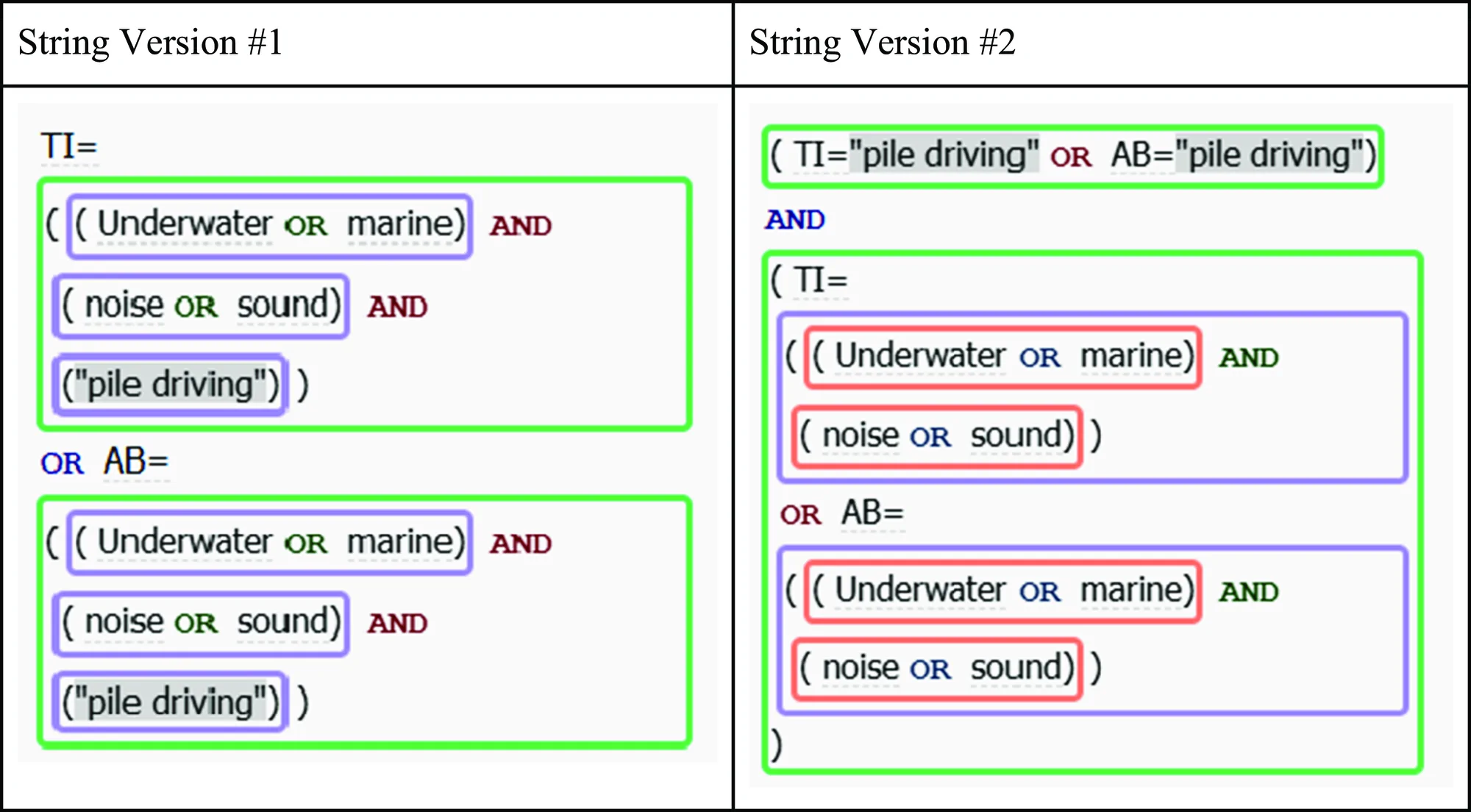
Two different search strings using different approaches to Boolean architecture are shown. String Version #1 is a simpler approach combining the three PEO concepts within each search field. String Version #2 shows a more complex approach with nested Boolean phrases for a more comprehensive search. This figure shows strings in Web of Science syntax (visualized in https://www.medsyntax.org). String syntax was adapted to meet the syntax requirements for each database. Full strings can be found in the supplementary file. Figure 3 Long description.

To demonstrate CiteSource’s application in search string development and optimization, this example examines different approaches to Boolean architecture for a simple search string. Due to differences in the way database platforms function, it is often useful to compare search techniques, including variations in Boolean nesting (i.e., the construction of parentheticals), metadata field selection, and verifying correct use of syntax. For example, some database platforms do not allow users to combine the results of multiple strings, nest Boolean logic, or combine field codes to search across multiple fields at once; consequently, simplified search strategies are often required to accommodate these limitations. In this example, our team is once again examining the impact of pile driving on marine life, utilizing a search string that represents three concepts: marine/underwater environment (population), pile driving (exposure), and noise/sound (outcome). Two search strings (Figure 3) were developed with their Boolean architecture intentionally designed to yield different results, illustrating CiteSource’s capability to facilitate analysis of such variations. The first version, used in the prior scenario, is designed for broad compatibility across most platforms, reflecting a common necessity due to database constraints. The second string involves nested Boolean logic, representing a more robust and strategic approach that can be used in databases like Web of Science. Here, Web of Science, Dimensions, and Scopus have been selected to demonstrate how CiteSource can compare search results across different string approaches and database platforms; while observing string variations might not always necessitate multiple platforms, applying this method here serves to further showcase CiteSource’s capabilities in cross-database analysis.

**Figure 4:**
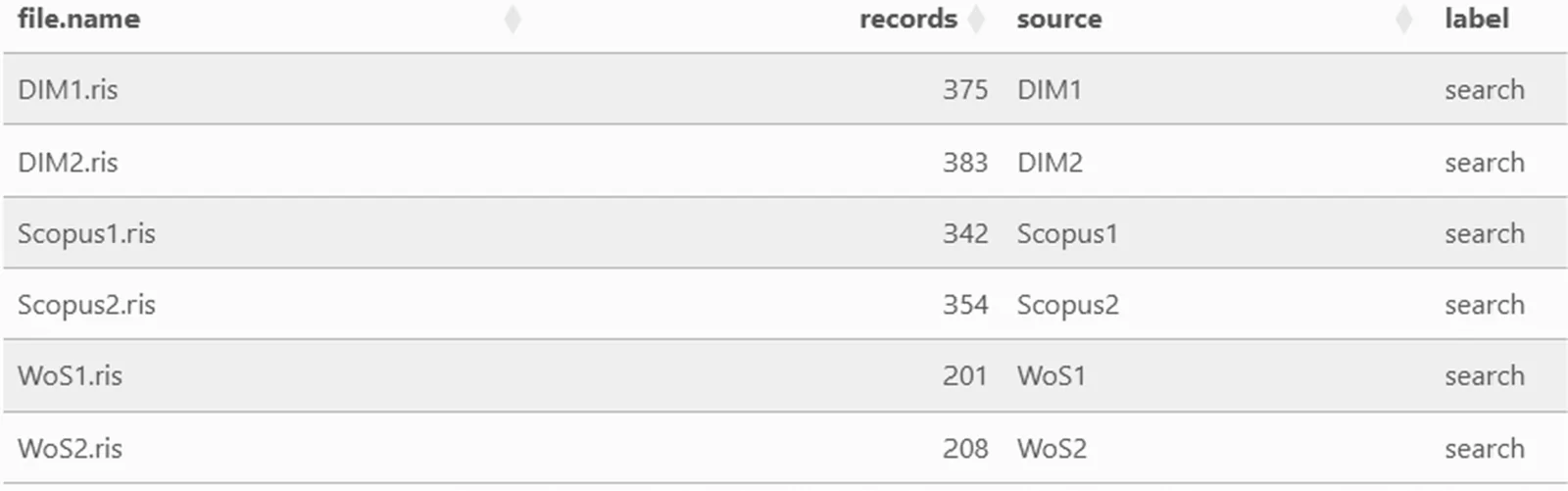
After uploading files, users have the ability to tag each accordingly. In this case, six uploaded .ris files are tagged according to the information source and string. For efficiency, CiteSource auto-fills the source field according to the file name and automatically adds ‘search’ for each label, as this is the most common tag.

**Figure 5:**
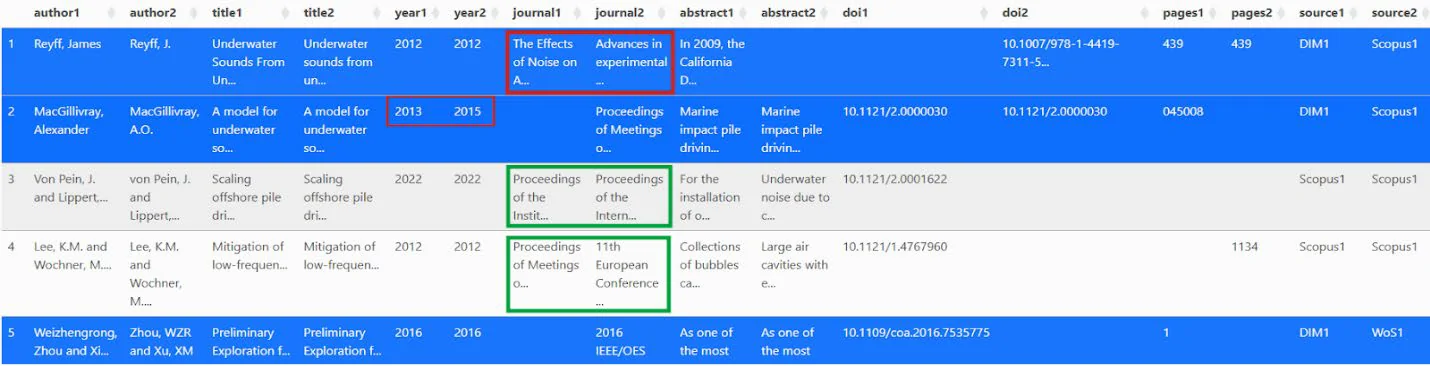
Manual deduplication table. The three rows highlighted in blue have been selected as duplicates. Columns show metadata for each record identified as a potential duplicate. Green boxes are added here to show records in pairs #3 and #4 are separate conference papers. Red boxes are added here to highlight incorrect metadata. Figure 5 Long description.

**Figure 6:**
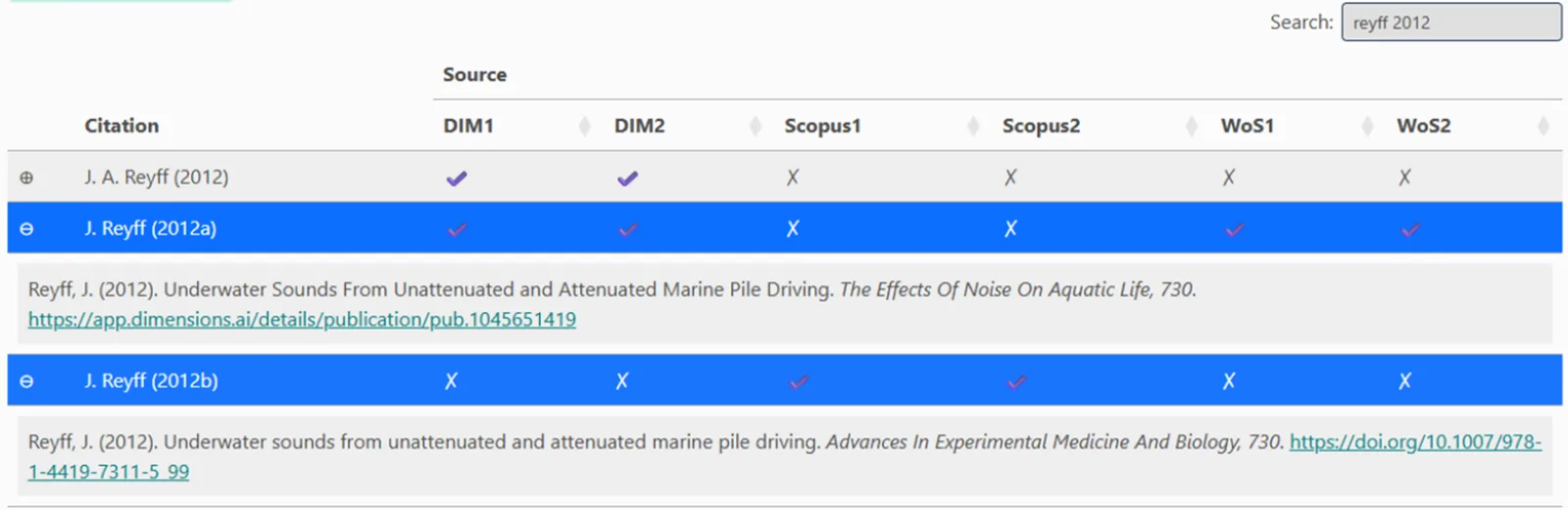
Individual record table is shown with two records that are identified in pair #1 from Figure 5. Used in conjunction with the manual deduplication table, records can be quickly checked by linking directly to the item. In this case, record ‘Reyff 2012a’ is correct, while metadata from 2012b incorrectly uses the book series title. Figure 6 Long description.

After importing the search result files for each string version and database, metadata tags are assigned within CiteSource to differentiate each distinct set of results (Figure 4). CiteSource streamlines tagging and helps reduce errors by optionally auto-filling the ‘source’ field based on the file name; it also defaults the ‘label’ field to ‘search’, the most common initial designation. Both the source and label fields can be easily edited as necessary. Once citation files are tagged, we perform automated and manual deduplication (Figure 5). To efficiently resolve these flagged pairs, CiteSource’s ‘Individual Record Table’ (Figure 6) is particularly useful, as it displays comprehensive bibliographic details for every record, and critically, provides direct links (e.g., via DOI or URL) to the original source documents when available. By examining the manual deduplication table in combination with the full details, users can quickly access the source articles and accurately determine if the flagged items are true duplicates, even in cases of inconsistent or incomplete metadata.

**Figure 7:**
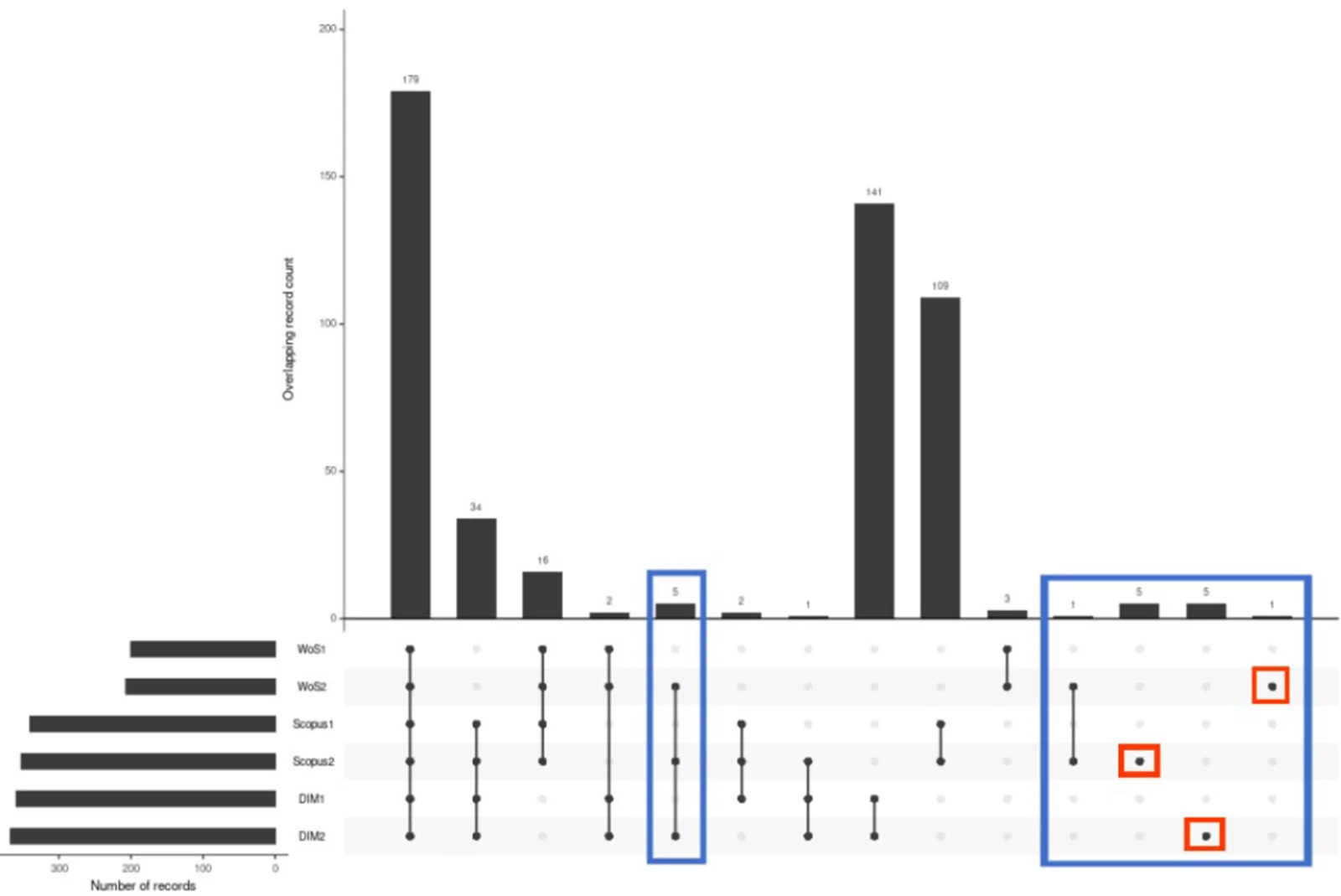
The upset plot visualization can be used to assess the number of overlapping and unique records across each search string version and across databases (WoS=Web of Science; DM=Dimensions). This upset plot shows 17 additional records being found using Search String Version #2; blue boxes are added to highlight these records. Of the 17 additional records, 11 are unique to one of the three sources; red boxes highlight these records. Figure 7 Long description.

**Figure 8:**
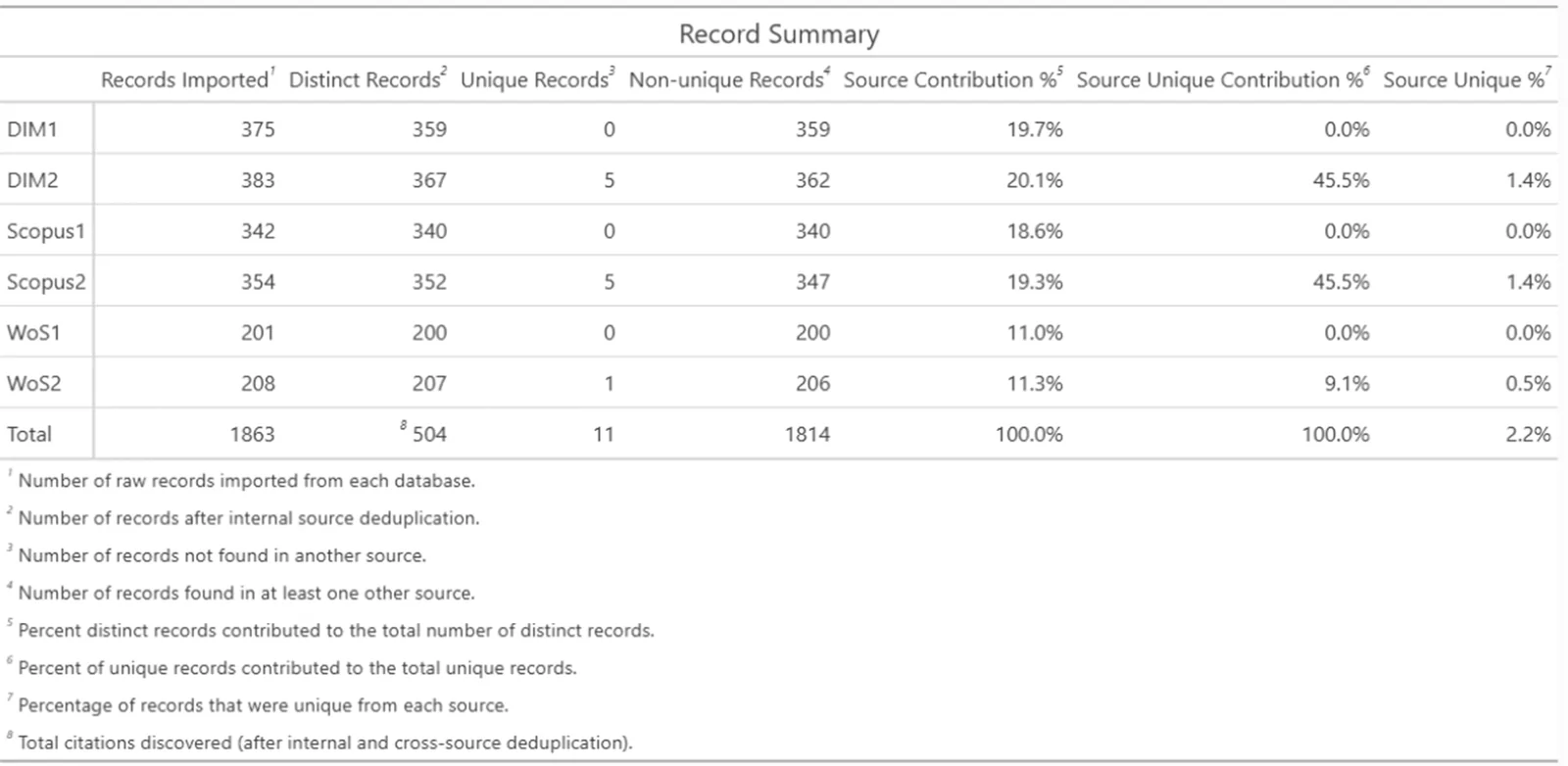
The Detailed Record Table provides information on the number of imported records, distinct records, unique records, duplicate records, the percentage of records contributed by each source, the percentage of unique records contributed by each source, and the percentage of each source that was unique. Figure 8 Long description.

CiteSource utilizes the UpsetR package^43^ to visualize overlap across sources (Figure 7). This plot offers a powerful visualization for comparing the retrieval of different search string versions across multiple databases. Each row represents an information source, defined by the user with the ‘source’ field. Columns show the number of records that overlap across these sources. In this example, examining the intersections reveals that String Version #2 identified 17 records not captured by String Version #1(blue boxes indicate records found exclusively by String Version #2). The Detailed Record Table (Figure 8) is another way users can evaluate this overlap. The table shows the breakdown of each source, its overall record contribution, and the percentage of unique record contributions. In this case, there were only 11 unique records, 5 each from Dimensions and Scopus, and a single unique record from Web of Science.

#### 3.1.3 #3 Benchmark testing

One way to understand a search string’s effectiveness is through benchmark testing. By starting with a set of articles that meet the stated inclusion criteria, researchers can gauge the sensitivity and effectiveness of search strings, identify potential changes to a search string to improve sensitivity, and understand the limitations of the search.^44^ These benchmarking functionalities are particularly useful in fields where certain key studies are considered foundational. Ensuring these foundational studies are captured by search strategies is critical for the credibility and completeness of a review. Here, we demonstrate how CiteSource can assist researchers when benchmark testing the results of searches.

**Figure 9:**
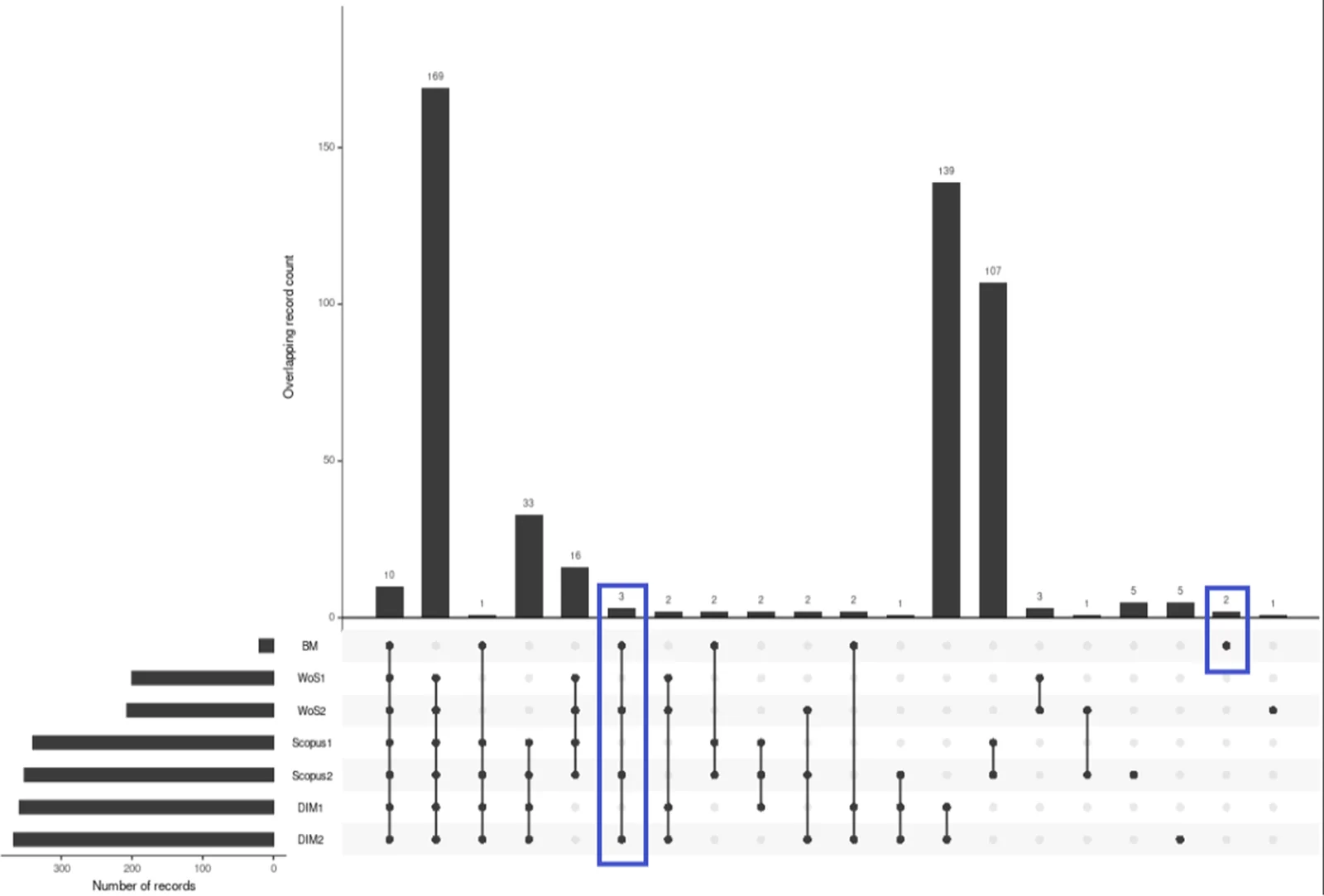
This upset plot shows an additional three benchmarking articles being found in Version #2 and two benchmarking articles that are not found. Blue boxes are added to highlight these records. Figure 9 Long description.

**Figure 10:**
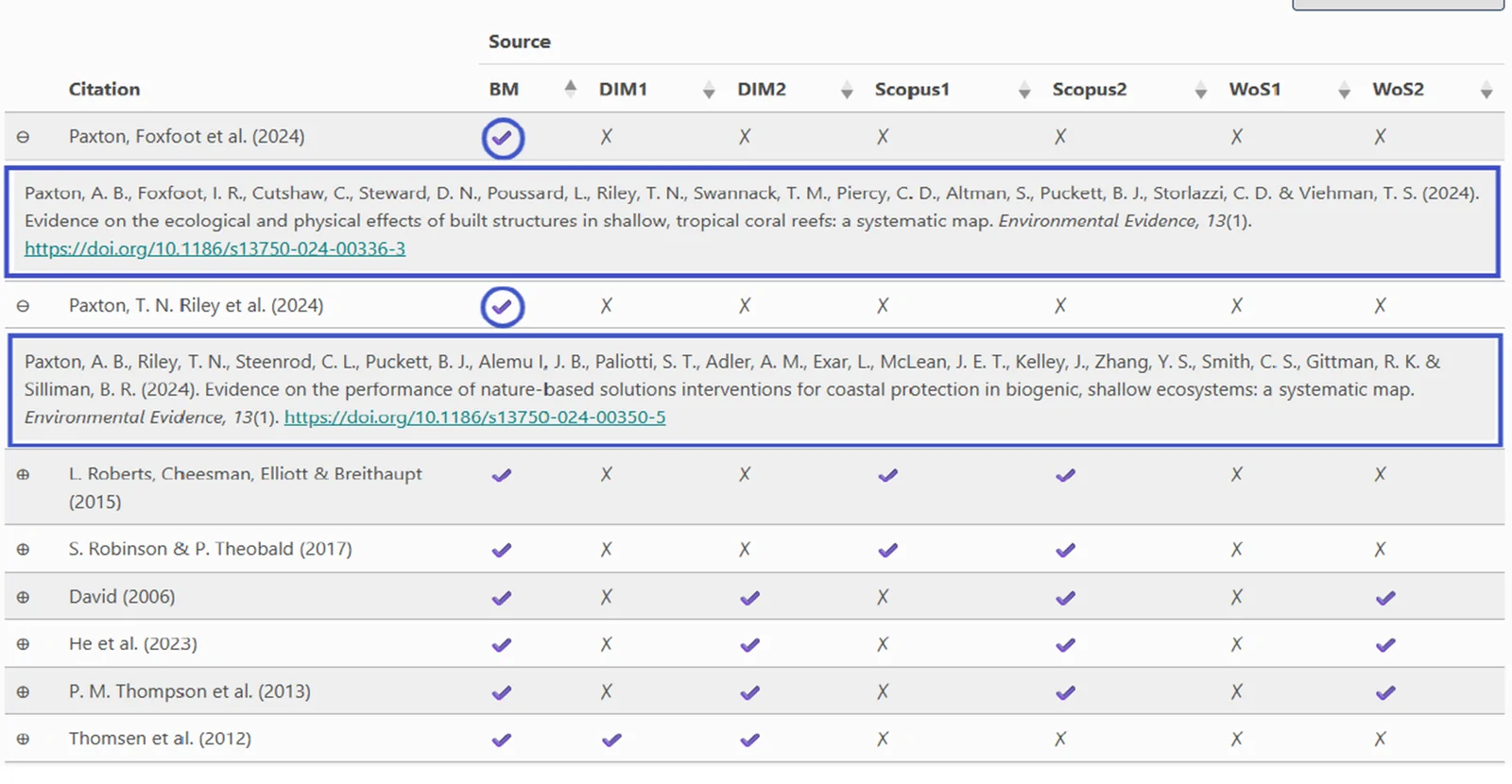
The individual record table facilitates the quick review of findings. In this example, we see the two benchmarking articles that were not found across the three sources using both string versions. Figure 10 Long description.

Continuing with the example above, we have hypothetically selected 20 articles as a benchmark set (designated ‘BM’) based on prior knowledge of the field and examining previous related reviews. In this case, the Upset plot (Figure 9 – similar to Figure 7 but now including the benchmark set as a distinct set) shows us the performance of our search strategies against these key articles. The plot reveals that ten of the 20 benchmarking articles (designated BM) were captured across the three databases by both String Version #1 and String Version #2. Looking at the remaining overlap, we can see that String Version #2 found three additional benchmark articles that String Version #1 missed, and that two benchmarking articles were not found in any database with either string. This information, used in combination with the record-level table (Figure 10), can be used to quickly review why these benchmark articles were missed, whether due to missing/incorrect terms/syntax, or whether these records were not indexed in the information sources.

### 3.2 Impact assessment

Transparent reporting of methods and results is a fundamental requirement once an evidence synthesis is complete, mandated by reporting guidelines and checklists.^45^ ^–^ ^47^ This includes details on search methods, information sources consulted, specific search strings utilized, and the flow of records through screening (often visualized in a PRISMA diagram). Beyond its utility in the iterative optimization of search strategies, CiteSource enables researchers to retrospectively analyze and quantify the contributions of different information sources and search methods to the resulting body of evidence. With minimal effort, this impact assessment analysis can increase transparency, empower future researchers to build upon documented effective practices, and contribute to refining methodological guidance within the evidence synthesis community.

#### 3.2.1 #4 Analyzing the impact of information sources & methods

**Figure 11:**
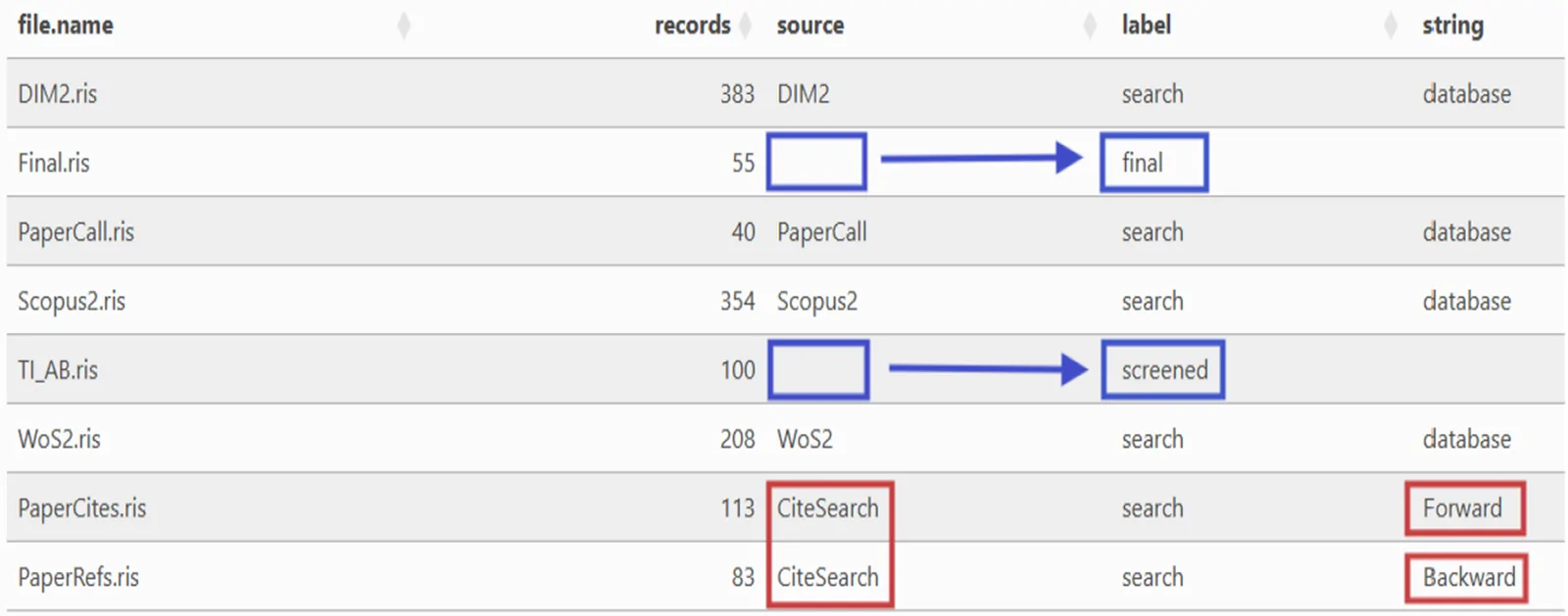
After uploading files to assess impact, users remove source tags for any files that represent records that have been screened and included in the final synthesis. Labels can be changed from ‘search’ to ‘screened’ or ‘final’. In this example, forward and backward citation chasing are combined as ‘CiteSearch’ in the ‘source’ field. Figure 11 Long description.

**Figure 12:**
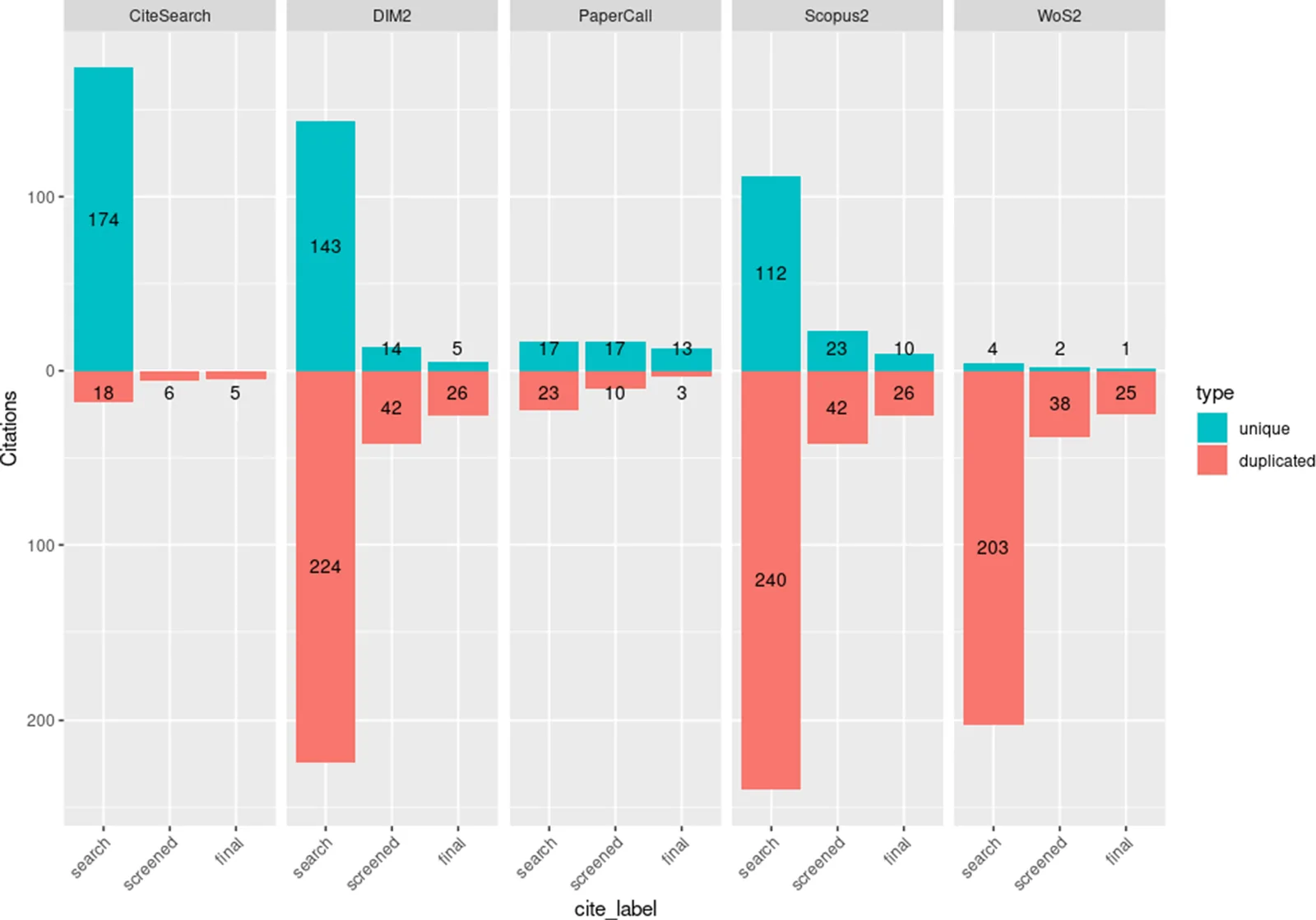
The phase analysis bar plot shows the number of unique and duplicate records across each source and screening phase. Each facet contains the data for an individual source. In this example, the call for papers yielded the lowest number of records, but had a significant number of unique articles that were included in the final synthesis. Figure 12 Long description.

**Figure 13:**
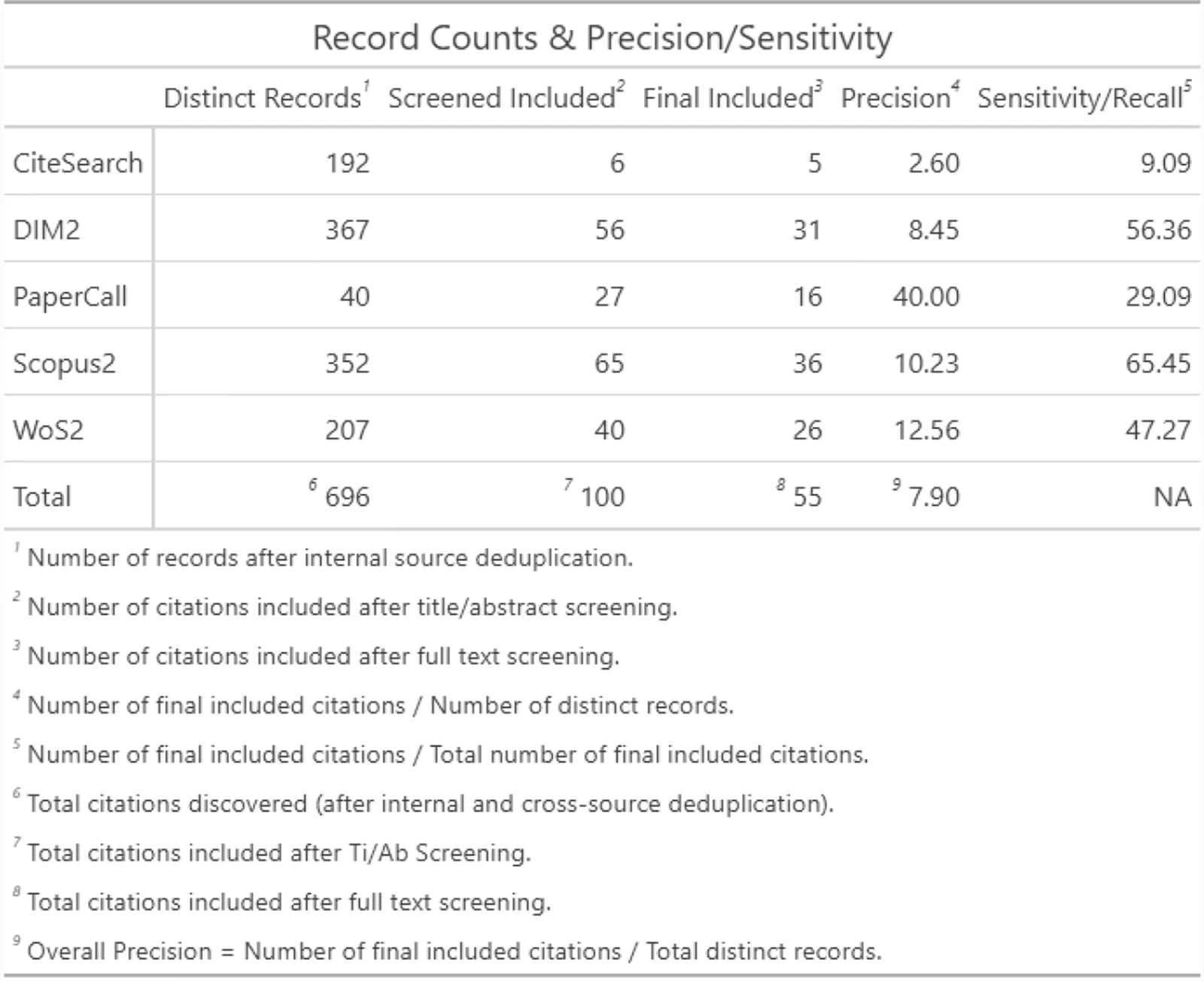
The precision/sensitivity table shows each source/method’s calculated precision and sensitivity. These calculations are based on the number of distinct records each source/method contributed, as well as the number of records from each source that were included in the final record set. Figure 13 Long description.

To illustrate how CiteSource can be used to analyze the impact of various sources and methods, we can return to the research team investigating acoustical disturbances from pile driving. Once the team completed their searches using their optimized string across Web of Science, Scopus, and Dimensions, they supplemented their search with a call for papers from experts in the field, as well as by applying forward and backward citation chasing on two seminal review articles. At title/abstract screening, the team includes 100 articles, and after full-text review, the resulting final set contains 55 articles. To analyze the impact of each source and method, the team can now upload all sets of records and tag them accordingly using the source and label fields (Figure 11). After automated and manual deduplication is complete, the team can generate the Phase Analysis plot (Figure 12), which provides a visual breakdown of each source’s unique/overlapping record count as well as a count of these records over the screening phases. Looking at the plot, it’s easy to see the performance of each information source and method. Citation searching, for example, while initially providing a large number of unique articles, contributed no unique records after title and abstract screening. In fact, this method was by far the least precise, as only five of the 192 distinct records it produced were included in the final synthesis. The plot also shows that while the call for papers yielded a small number of results, this method of discovery contributed the largest number of unique records to the final synthesis. Finally, we can see that both Scopus and Dimensions outperformed Web of Science in the number of unique records that they contributed to the final synthesis. The data are further detailed in the Precision/Sensitivity Table (Figure 13), providing a full picture of performance. Looking at the table, we can see that despite the lower number of unique articles included in the final synthesis, Web of Science had a slightly higher precision.

In practical terms, this impact assessment functionality can lead to more strategic decision-making in resource selection and method application. This quantification of contribution and overlap builds upon methods such as the Search Summary Table (SST) devised by Alison Bethel, which offers a structured overview of search results, indicating which searches found included references and providing sensitivity and precision calculations for search methods.^48^ For instance, if certain methods consistently yield high-quality results that pass through all screening phases, they might be prioritized in future reviews or updates to the review. Conversely, methods or sources that contribute little value can be de-emphasized or excluded. This functionality enhances transparency about where and how included studies were found, facilitates a data-driven approach for future search method decisions, and deepens the understanding of the true impact of different resources and search methods. By providing insights into the performance and impact of each resource or method, CiteSource ensures that systematic reviews are both comprehensive and methodologically sound, supporting more robust and credible evidence synthesis.

## 4 Implications for evidence synthesis

CiteSource improves the rigor and effectiveness of evidence synthesis by providing data to inform critical decisions made during the search process. Traditionally, search strategy development often relies heavily on the researcher’s experience, knowledge, and sometimes outdated guidance. CiteSource moves beyond this by allowing researchers to compare and optimize various aspects of their search strategy during the search strategy development phase.

**Enhanced Efficiency and Time Savings:** Evidence synthesis is time-consuming, especially the iterative process of developing and validating search strategies. CiteSource significantly compresses this cycle by offering rapid analysis and visualization (Heatmaps, Upset Plots, and Summary Tables) immediately after deduplication. This allows researchers to test variations in sources, strings, and methods much more quickly than analyzing results individually, saving time that can be redirected to critical analysis and synthesis. For example, in the case of benchmarking, if a researcher wanted to analyze three variations of a string across three databases using ten benchmarking articles, each of the nine results would need to be cross-referenced against the set of benchmarking articles. This would require the user to search for each of the ten articles nine times and document the results. With CiteSource, the researcher can quickly analyze all nine sets of results alongside the benchmarking articles to see which articles were found using each string across each database. These results can also be exported in .csv or .ris for later reference and reporting.

**Data-Driven Decisions for Optimization:** CiteSource enables researchers to use data to make informed decisions about which information sources provide the best return on investment by quantifying their unique contributions and overlap. It also facilitates the testing and refinement of search strings by allowing rapid comparison of different variations and their impact on retrieval. This allows for a more strategic approach to balancing sensitivity and precision. Benchmark testing is also more feasible with CiteSource, as it provides a quantitative way to assess if key articles are captured.

**Enhanced Reporting and Transparency:** Reporting guidelines like PRISMA and ROSES require transparent and detailed documentation of the search process. CiteSource directly supports reporting by generating clear outputs and maintaining provenance. Plots and tables offer visual and quantitative summaries. Crucially, the tool embeds custom metadata into standard fields during export, providing a clear and reproducible audit trail of the methodology. This enhances the credibility and reproducibility of the synthesis.

**Support for Methods Research:** By providing a platform for quantifying the performance of different sources and methods (e.g., through precision/sensitivity calculations), CiteSource exposes underlying data such as detailed overlap statistics, source contribution breakdowns, and precision/sensitivity metrics across different search iterations, which can inform the future development of evidence-based search guidelines for the wider community. CiteSource also supports methodological research on searching itself, potentially facilitating ‘Study Within A Review’ (SWAR) focused on search methodology,^49^ speed analysis of sources and methods,^17^ ^,^ ^20^ ^,^ ^50^ ^–^ ^55^ and support initiatives and frameworks to improve search strategy development and validation, such as searchRxiv and CEEDER.^56^ ^–^ ^60^

## 5 Application beyond evidence synthesis

Beyond its direct contributions to the evidence synthesis workflow, CiteSource also offers valuable applications in related areas such as training and resource management.

**Training and Education:** CiteSource serves as an effective training tool for students and early-career researchers learning evidence synthesis methods. Its interactive visualizations offer a hands-on way to understand abstract concepts like Boolean logic, database coverage, and effects of string variations on search outcomes. Instructors can use it to demonstrate best practices and build practical skills in systematic searching. This makes the learning process more intuitive and data-informed.

**Library Collection Development:** Librarians, often tasked with making difficult resource allocation decisions, can leverage CiteSource to inform their collection development strategies. By analyzing search results, they can gather objective data on database coverage and overlap for specific research topics that are critical to their patrons. These data can be used to justify subscription costs, compare existing resources with potential new ones, and make recommendations based on demonstrated value and uniqueness.

## 6 Conclusion

The CiteSource R package and Shiny application provide a foundation for a new era of data-driven search strategy design in evidence synthesis. As the information landscape continues to evolve, openly available and accessible tools like CiteSource will be crucial for empowering researchers to make informed decisions. CiteSource’s unique ability to enhance transparency in reporting, support aggregate analysis of search performance, and expedite the iterative design and validation processes provides immediate, tangible benefits for improving the efficiency and rigor of evidence gathering. However, the true potential of CiteSource to transform the field will be realized through its widespread adoption and the collective contribution of its users. With regular reporting of these data, research teams have an opportunity to collectively contribute findings that can be further synthesized, leading to enhanced methodological practices and more robust, evidence-based guidelines for search strategy development. Whether integrated into the development of new syntheses to optimize evidence discovery or applied retrospectively to analyze and learn from past projects, CiteSource offers a powerful means to strengthen the quality, defensibility, and methodological integrity of evidence synthesis methods.

## Data Availability

The source code of the software is publicly available on GitHub (https://github.com/ESHackathon/CiteSource). All citation data presented in this paper are available on OSF (https://osf.io/hxusf/overview).

## Long descriptions

### Table 1 Long description

The table consists of two columns: Plots and Function.

* Row 1: Heatmaps. Function: Visualize the number and percent of overlap between two sources, methods, or techniques.

* Row 2: Upset plot. Function: Visualize the number of unique and overlapping records across multiple sources, methods, or techniques.

* Row 3: Bar chart. Function: Visualize the number of unique and overlapping records for each source, method, or technique across screening phases.

Navigate back to Table 1.

### Table 2 Long description

The table consists of two columns: Tables and Function.

* Row 1: The Record summary table displays the number of unique or overlapping records, the percent of unique records contributed, and the percent of records unique to each source, method, or technique.

* Row 2: The Precision sensitivity table provides an overview of the number of records included across screening phases for each source or method. It includes precision and sensitivity or recall calculations for each.

* Row 3: The Record level table is an interactive table allowing users to search full citations, sort based on source or method association, and link directly to records via D O I or U R L.

Navigate back to Table 2.

### Figure 2 Long description

The heatmap uses a blue color scale where darker shades represent higher record counts, ranging from 0 to over 300.

Row 1, D I M 1: Intersects with itself at 361.

Row 2, Scopus 1: Intersects with D I M 1 at 212 and itself at 341.

Row 3, W o S 1: Intersects with D I M 1 at 181, Scopus 1 at 193, and itself at 200.

Row 4, A S F A 1: Intersects with D I M 1 at 56, Scopus 1 at 67, W o S 1 at 55, and itself at 96.

Row 5, O A 1: Intersects with D I M 1 at 51, Scopus 1 at 61, W o S 1 at 50, A S F A 1 at 77 (highlighted with an orange border), and itself at 77.

Row 6, O A T D 1: Intersects with D I M 1, Scopus 1, W o S 1, A S F A 1, and O A 1 all at 0 (these five cells are enclosed in a green rectangular border). It intersects with itself at 16.

A legend on the right indicates the ‘Records’ scale from 0 (white) to 300 (dark blue).

Navigate back to Figure 2.

### Figure 3 Long description

The diagram consists of two panels labeled String Version number 1 and String Version number 2.

Panel 1 (Left): String Version number 1.

This panel shows two identical green boxes separated by the operator O R.

- The top green box starts with T I equals. Inside, three purple boxes are stacked vertically and joined by A N D operators. The first purple box contains Underwater O R marine. The second contains noise O R sound. The third contains the phrase pile driving in quotes.

- The bottom green box starts with A B equals and contains the exact same three purple boxes and A N D logic as the top box.

Panel 2 (Right): String Version number 2.

This panel shows a more complex nested structure.

- At the top is a green box containing T I equals pile driving O R A B equals pile driving.

- Below this is an A N D operator followed by a large green box containing two purple boxes separated by an O R operator.

- The first purple box starts with T I equals. Inside are two orange boxes joined by A N D. The first orange box contains Underwater O R marine and the second contains noise O R sound.

- The second purple box starts with A B equals and contains the same two orange boxes and A N D logic as the purple box above it.

Navigate back to Figure 3.

### Figure 5 Long description

The table contains 16 columns: author 1, author 2, title 1, title 2, year 1, year 2, journal 1, journal 2, abstract 1, abstract 2, D O I 1, D O I 2, pages 1, pages 2, source 1, and source 2.

* Row 1: Highlighted in blue. A red box surrounds journal 1 ‘The Effects of Noise on A...’ and journal 2 ‘Advances in experimental...’, indicating a metadata mismatch.

* Row 2: Highlighted in blue. A red box surrounds year 1 ‘2013’ and year 2 ‘2015’, indicating a metadata mismatch.

* Row 3: White background. A green box surrounds journal 1 ‘Proceedings of the Instit...’ and journal 2 ‘Proceedings of the Intern...’, identifying these as separate conference papers rather than duplicates.

* Row 4: White background. A green box surrounds journal 1 ‘Proceedings of Meetings o...’ and journal 2 ‘11th European Conference...’, identifying these as separate conference papers.

* Row 5: Highlighted in blue. Shows matching metadata for authors Weizhengrong, Zhou and Xi and Zhou, W Z R and Xu, X M, with matching D O I 10.1109/coa.2016.7535775.

Navigate back to Figure 5.

### Figure 6 Long description

The table features a search bar at the top right containing the text reyff 2012. Below this, a header row lists Citation followed by Source categories: D I M 1, D I M 2, Scopus 1, Scopus 2, W o S 1, and W o S 2.

* The first row shows J. A. Reyff (2012) with checkmarks for D I M 1 and D I M 2, and X marks for Scopus and W o S columns.

* The second row, highlighted in blue, shows J. Reyff (2012a). It has checkmarks for D I M 1, D I M 2, W o S 1, and W o S 2, with X marks for Scopus 1 and Scopus 2. Below this row is the full citation: Reyff, J. (2012). Underwater Sounds From Unattenuated and Attenuated Marine Pile Driving. The Effects Of Noise On Aquatic Life, 730, followed by a D I M E N S I O N S dot A I U R L.

* The third row, also highlighted in blue, shows J. Reyff (2012b). It has checkmarks for Scopus 1 and Scopus 2, with X marks for all D I M and W o S columns. Below this row is the full citation: Reyff, J. (2012). Underwater sounds from unattenuated and attenuated marine pile driving. Advances In Experimental Medicine And Biology, 730, followed by a D O I dot O R G U R L.

Navigate back to Figure 6.

### Figure 7 Long description

The visualization consists of three main components.

1. Bottom-Left Horizontal Bar Chart: Labeled Number of records, it shows the total size for six sets. From top to bottom: W o S 1 (approximately 200), W o S 2 (approximately 210), Scopus 1 (approximately 320), Scopus 2 (approximately 330), D I M 1 (approximately 340), and D I M 2 (approximately 350).

2. Bottom-Right Matrix: A grid of dots and lines indicating intersections between the six sets.

* A blue vertical rectangle highlights a column where W o S 2, Scopus 2, and D I M 2 intersect.

* A large blue square on the far right highlights four columns representing records found in Version 2 but not Version 1.

* Within this blue square, three specific single-dot intersections (W o S 2 only, Scopus 2 only, and D I M 2 only) are outlined in red boxes to indicate unique records.

3. Top Vertical Bar Chart: Labeled Overlapping record count, it shows the size of each intersection corresponding to the matrix columns below. From left to right, the largest bars are:

* 179 records (intersection of all six sets).

* 141 records (intersection of Scopus 1, Scopus 2, D I M 1, and D I M 2).

* 109 records (intersection of Scopus 2 and D I M 2).

* The blue-highlighted column in the middle shows 5 records.

* The four columns in the far-right blue square show counts of 1, 5, 5, and 1 respectively.

Navigate back to Figure 7.

### Figure 8 Long description

The table consists of 8 rows and 8 columns. The columns are labeled: Source Name, Records Imported super 1, Distinct Records super 2, Unique Records super 3, Non-unique Records super 4, Source Contribution % super 5, Source Unique Contribution % super 6, and Source Unique % super 7.

Data rows:

* D I M 1: 375, 359, 0, 359, 19.7%, 0.0%, 0.0%.

* D I M 2: 383, 367, 5, 362, 20.1%, 45.5%, 1.4%.

* Scopus 1: 342, 340, 0, 340, 18.6%, 0.0%, 0.0%.

* Scopus 2: 354, 352, 5, 347, 19.3%, 45.5%, 1.4%.

* W o S 1: 201, 200, 0, 200, 11.0%, 0.0%, 0.0%.

* W o S 2: 208, 207, 1, 206, 11.3%, 9.1%, 0.5%.

* Total: 1863, super 8 504, 11, 1814, 100.0%, 100.0%, 2.2%.

Footnotes:

1. Number of raw records imported from each database.

2. Number of records after internal source deduplication.

3. Number of records not found in another source.

4. Number of records found in at least one other source.

5. Percent distinct records contributed to the total number of distinct records.

6. Percent of unique records contributed to the total unique records.

7. Percentage of records that were unique from each source.

8. Total citations discovered after internal and cross-source deduplication.

Navigate back to Figure 8.

### Figure 9 Long description

The top section is a vertical bar chart titled Overlapping record count on the y-axis, ranging from 0 to 150. Bars represent intersection sizes, with the largest peaks at 169, 139, and 107. A blue box highlights a bar with a count of 3, and another blue box highlights a bar with a count of 2 near the right end of the x-axis.

The bottom-left section contains horizontal bars showing the total Number of records for each set: B M (smallest), W o S 1, W o S 2, Scopus 1, Scopus 2, D I M 1, and D I M 2 (largest, exceeding 300).

The bottom-right section is an intersection matrix. Rows correspond to the sets and columns correspond to the vertical bars above. Black dots connected by vertical lines indicate which sets are included in an intersection.

* The first blue box highlights a column where dots are present for W o S 2, Scopus 2, D I M 1, and D I M 2, resulting in the count of 3.

* The second blue box highlights a column with a single dot for the B M set, resulting in the count of 2.

Navigate back to Figure 9.

### Figure 10 Long description

The table consists of a header row and eight data rows. The columns are labeled Citation, B M, D I M 1, D I M 2, Scopus 1, Scopus 2, W o S 1, and W o S 2.

* The first row shows Paxton, Foxfoot et al. 2024 with a circled checkmark in the B M column and X marks in all other columns. An expanded sub-row provides the full bibliographic reference and a D O I link.

* The second row shows Paxton, T. N. Riley et al. 2024 with a circled checkmark in the B M column and X marks in all other columns. An expanded sub-row provides the full bibliographic reference and a D O I link.

* The third row for L. Roberts et al. 2015 shows checkmarks in B M, Scopus 1, and Scopus 2, with X marks in the remaining columns.

* The fourth row for S. Robinson and P. Theobald 2017 shows checkmarks in B M, Scopus 1, and Scopus 2, with X marks in the remaining columns.

* The fifth row for David 2006 shows checkmarks in B M, D I M 2, Scopus 2, and W o S 2, with X marks in the remaining columns.

* The sixth row for He et al. 2023 shows checkmarks in B M, D I M 2, Scopus 2, and W o S 2, with X marks in the remaining columns.

* The seventh row for P. M. Thompson et al. 2013 shows checkmarks in B M, D I M 2, Scopus 2, and W o S 2, with X marks in the remaining columns.

* The eighth row for Thomsen et al. 2012 shows checkmarks in B M, D I M 1, and D I M 2, with X marks in the remaining columns.

Navigate back to Figure 10.

### Figure 11 Long description

The table contains five columns.

Row 1: file dot name D I M 2 dot ris, 383 records, source D I M 2, label search, string database.

Row 2: file dot name Final dot ris, 55 records. A blue arrow points from an empty source box to a blue-outlined label box containing the word final.

Row 3: file dot name PaperCall dot ris, 40 records, source PaperCall, label search, string database.

Row 4: file dot name Scopus 2 dot ris, 354 records, source Scopus 2, label search, string database.

Row 5: file dot name T I underscore A B dot ris, 100 records. A blue arrow points from an empty source box to a blue-outlined label box containing the word screened.

Row 6: file dot name W o S 2 dot ris, 208 records, source W o S 2, label search, string database.

Row 7: file dot name PaperCites dot ris, 113 records. The source CiteSearch and the string Forward are both outlined in red boxes. The label is search.

Row 8: file dot name PaperRefs dot ris, 83 records. The source CiteSearch and the string Backward are both outlined in red boxes. The label is search.

Navigate back to Figure 11.

### Figure 12 Long description

The plot consists of five vertical panels labeled CiteSearch, D I M 2, PaperCall, Scopus 2, and W o S 2. The Y axis represents Citations, ranging from 200 below zero to 100 above zero. The X axis for each panel lists three phases: search, screened, and final. Teal bars extending upward represent unique records, while coral bars extending downward represent duplicated records.

* CiteSearch: Search phase has 174 unique and 18 duplicated. Screened and final phases have 0 unique and 6 and 5 duplicated respectively.

* D I M 2: Search phase has 143 unique and 224 duplicated. Screened has 14 unique and 42 duplicated. Final has 5 unique and 26 duplicated.

* PaperCall: Search phase has 17 unique and 23 duplicated. Screened has 17 unique and 10 duplicated. Final has 13 unique and 3 duplicated.

* Scopus 2: Search phase has 112 unique and 240 duplicated. Screened has 23 unique and 42 duplicated. Final has 10 unique and 26 duplicated.

* W o S 2: Search phase has 4 unique and 203 duplicated. Screened has 2 unique and 38 duplicated. Final has 1 unique and 25 duplicated.

A legend on the right identifies teal as unique and coral as duplicated.

Navigate back to Figure 12.

### Figure 13 Long description

The table contains six columns: Source, Distinct Records super 1, Screened Included super 2, Final Included super 3, Precision super 4, and Sensitivity or Recall super 5.

Data rows:

* CiteSearch: 192, 6, 5, 2.60, 9.09.

* D I M 2: 367, 56, 31, 8.45, 56.36.

* PaperCall: 40, 27, 16, 40.00, 29.09.

* Scopus 2: 352, 65, 36, 10.23, 65.45.

* W o S 2: 207, 40, 26, 12.56, 47.27.

* Total: 696 super 6, 100 super 7, 55 super 8, 7.90 super 9, N A.

Footnotes:

1. Number of records after internal source deduplication.

2. Number of citations included after title or abstract screening.

3. Number of citations included after full text screening.

4. Number of final included citations / Number of distinct records.

5. Number of final included citations / Total number of final included citations.

6. Total citations discovered after internal and cross-source deduplication.

7. Total citations included after T i / A b Screening.

8. Total citations included after full text screening.

9. Overall Precision = Number of final included citations / Total distinct records.

Navigate back to Figure 13.
